# Supplementary material for: The interactome of intact mitochondria by cross-linking mass spectrometry provides evidence for coexisting respiratory supercomplexes
Source: Mol Cell Proteomics. 2017 Dec 8;17(2):216–32. doi: 10.1074/mcp.RA117.000470 (PMC5795388; doi:10.1074/mcp.RA117.000470)
Supplement: Supplemental Data [file supp_RA117.000470_133922_0_supp_23978_fzffwf.zip › spectra_annotation/mito_DR_spectra_annotation/369-1-3-1-21-1.pdf]

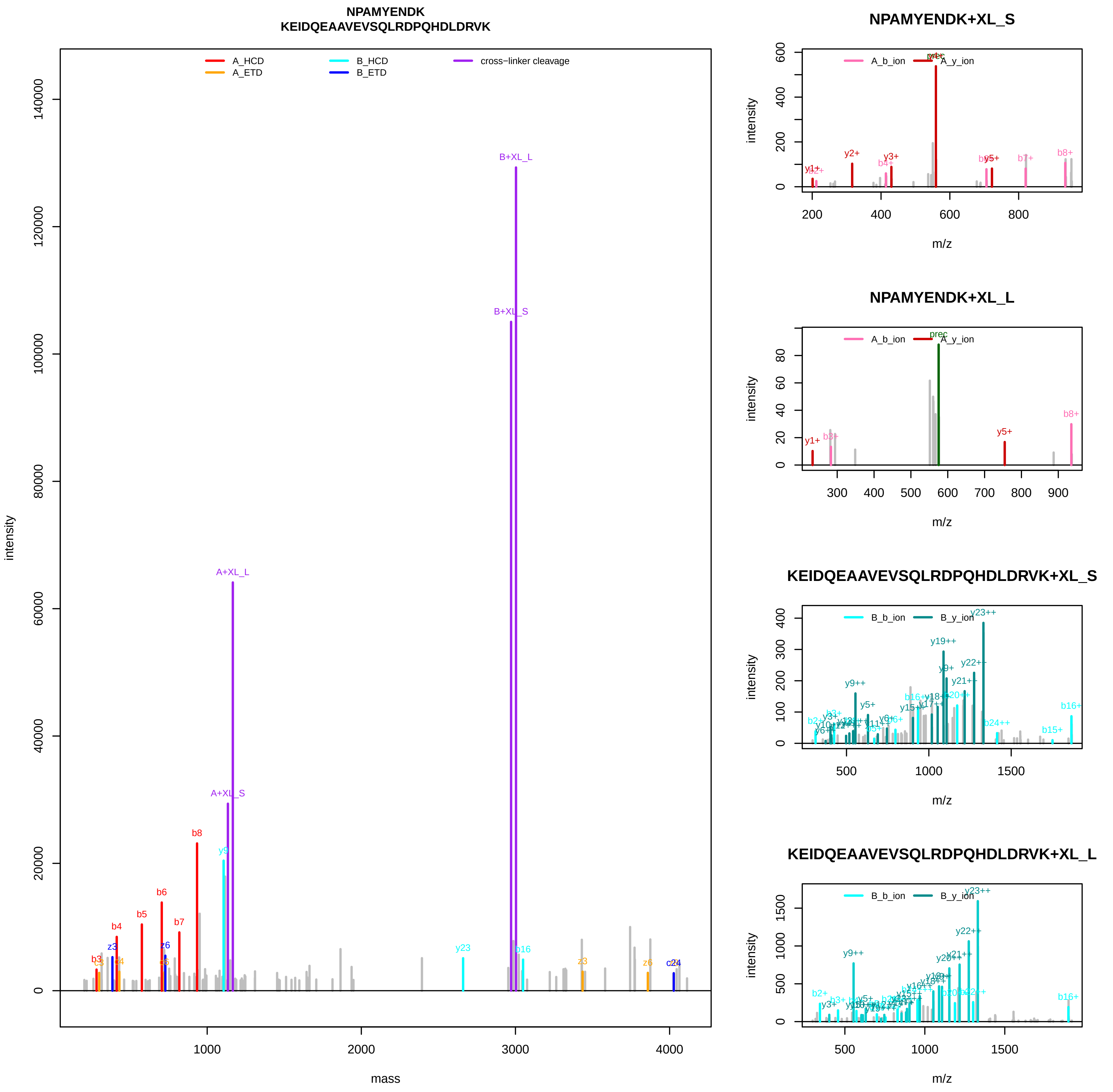
NPAMYENDK
KEIDQEAAVEVSQLRDPQHDLDRVK
A_HCD
A_ETD
B_HCD
B_ETD
cross−linker cleavage
B+XL_L
B+XL_S
A+XL_L
A+XL_S
b3
b4
b5
b6
b7
b8
z3
z6
y9
y23
b16
z3
z6
c24
intensity
mass
0
20000
40000
60000
80000
100000
120000
140000
1000
2000
3000
4000
NPAMYENDK+XL_S
A_b_ion
A_y_ion
prec
y1+
y2+
y3+
b4+
y5+
b7+
b8+
intensity
m/z
NPAMYENDK+XL_L
y1+
b3+
y5+
b8+
KEIDQEAAVEVSQLRDPQHDLDRVK+XL_S
B_b_ion
B_y_ion
y23++
y19++
y22++
y9++
y21++
y5+
b16+
b24++
b15+
KEIDQEAAVEVSQLRDPQHDLDRVK+XL_L
y23++
y22++
y9++
y21++
y20++
b2+
b16+
